# Observational evidence that economic reciprocity pervades self-organized food co-operatives

**DOI:** 10.1017/ehs.2025.8

**Published:** 2025-02-14

**Authors:** Taylor Z. Lange, Timothy M. Waring

**Affiliations:** 1 Margaret Chase Smith Policy Center, University of Maine, Orono, ME, USA; 2 Ecology and Environmental Sciences, University of Maine, Orono, ME, USA; 3 School of Economics, University of Maine, Orono, ME, USA; 4 Mitchell Center for Sustainability Solutions, University of Maine, Orono, ME, USA

**Keywords:** reciprocity, cooperation, coordination, collective action, cooperatives, food-buying clubs

## Abstract

Evolutionary scientists argue that prosociality has been central to human ecological success. Theoretical models and behavioural experiments have found that prosociality, and cooperation in particular, is conditional and context dependent, that individuals vary in their propensity to cooperate, and that reciprocity stabilizes these behaviours within groups. Experimental findings have had limited validation with observations of behaviour in natural settings, especially in organizational contexts. Here, we report *in situ* measurements of collective action, which show that reciprocity is abundant in organizations embedded in a cash economy. We study small ‘food clubs’, where members share bulk purchases and are considered to be heavily dependent on cooperation. We use high-resolution data on the economic interactions of 1,528 individuals across 35 clubs and over a combined 107 years of operation. We develop a network method to detect different directional and temporal forms of economic reciprocity, and statistically classify individual behavioural types akin to those in experiments. We find abundant direct reciprocity, supplemented by indirect reciprocity, and that members of most clubs can be identified as consistent reciprocators. This study provides initial observational evidence that economic reciprocity may be more abundant in real-world settings, sharpening the findings of the behavioural study of cooperation and contributing to the more naturalistic study of reciprocity and prosociality.

## 1. Introduction

Humans and other social organisms face a variety of social dilemmas where the interests of the group may be at odds with those of the individual, and the human ability to solve these dilemmas is theorized to be central to our success as a species (Gowdy, 2021; Gowdy & Krall, 2016; Henrich, 2015). In collective action situations, where a group works towards a common goal, individuals are often required to act prosocially and disregard the incentive to free-ride off the efforts of others (Fischbacher & Gächter, 2010; Fischbacher et al., 2001; Gintis, 2009). Theoretical models, laboratory experiments, and field experiments have revealed several mechanisms that stabilize individual prosociality by minimizing cheating, with reciprocity accumulating the most substantial amount of scholarship (Axelrod & Hamilton, 1981; Nowak, 2006; Nowak & Sigmund, 2005; Ohtsuki et al., 2006; Rand et al., 2014; Trivers, 1971). While these findings have been replicated across cultural contexts (Apicella et al., 2012; Henrich et al., 2001; Henrich & Muthukrishna, 2021; Kocher et al., 2008), observations of the patterns of reciprocity in non-experimental, contemporary contexts is lacking, leaving current findings circumscribed. Here, we present a novel study of collective action in organizational networks using observational data and comparing them to experimental and theoretical findings.

Prosocial behaviours are those that benefit others: they include coordination, cooperation, and altruism (Gintis, 2009; Henrich & Muthukrishna, 2021; Jensen, 2016). Coordination is the broadest category; it is defined as an action that benefits the individual and those around them (Gintis, 2009). Cooperation is generally understood as a person’s giving assistance to others in a manner that could still benefit that person, but which requires them to incur a personal cost (Henrich & Muthukrishna, 2021). Altruistic behaviours constitute a subset of cooperation and are defined as lending assistance in a manner that is not personally beneficial and also incurs a personal cost (Trivers, 1971; Wilson, 2015).

Reciprocity, or giving in equal measure to what one receives, is a potent behavioural strategy that sustains within-group prosociality across many experimental and theoretical collective action dilemmas (Janssen et al., 2011; Nowak, 2006; Pfeiffer et al., 2005; Sandroni, 2000; Schmid et al., 2021). In economic games with repeated interactions between two individuals such as the iterated prisoners’ dilemma, tit-for-tat direct reciprocity, i.e. doing as one’s partner did in the previous round, is the most successful strategy for sustaining cooperation and altruism specifically (Axelrod & Hamilton, 1981; Roberts, 2008). In another version of the iterated prisoners’ dilemma where partnerships change each round, pay-it-forward style indirect reciprocity allows cooperation to persist when (1) players can view their partner’s previous decisions and when (2) reputation accumulates over time (Nowak & Sigmund, 2005; Roberts, 2008). In public goods scenarios where individuals interact with many group members, reciprocity takes a more general form of ‘conditional cooperation’, where one gives the same as the rest of the group does on average (Fischbacher et al., 2001). However, free riders in public goods experiments can drag the average donation down, causing conditional cooperation to decline over time (Fischbacher et al., 2001; Thöni & Volk, 2018). Under such circumstances, cooperative behaviour needs additional supporting mechanisms such as interpersonal reward or punishment to coerce others into cooperation (Fischbacher & Gächter, 2010; Rand et al., 2009).

Many studies of conditional cooperation also suggest that individuals exhibit patterns of behaviour that can be classified into three broad types: conditional cooperators (whose cooperation is contingent on the cooperation of the rest of the group), altruists (who always cooperate), and free riders (who always defect) (Andreozzi et al., 2020; Fischbacher et al., 2001; Frey, 2017; Volk et al., 2012). These types are descriptions of individuals’ sustained strategy profiles throughout iterated games (Fischbacher et al., 2001) and they have been robustly replicated in person (Andreozzi et al., 2020; Deversi et al., 2020; Kocher et al., 2008) and in online video games (Frey, 2017, 2019). Furthermore, these types appear to be consistent when they participate in multiple experiments across time (Andreozzi et al., 2020; Volk et al., 2012), and they emerge in studies across cultural contexts (Frey, 2017; Kocher et al., 2008).

While these findings have contributed greatly to theories of the evolution and mechanisms of sustained cooperation (Nowak, 2006), tests of these theories with granular naturalistic data have remained elusive yet necessary, as experimental conditions often differ considerably from those in the real world. For example, experimental subjects are often drawn from large anonymous populations such as university student pools in which peers are strangers with no prior affiliation (e.g. Fischbacher et al., 2001 or Andreozzi et al., 2020) or are elicited through online services (e.g. Rand et al., 2009 or, 2014). Furthermore, the economic games played are game-theoretically derived to involve clearly differentiable cooperative or selfish decisions (Gintis, 2009). In contrast, most studies of real-world social dilemmas, including those surrounding public and similar goods, find that the individuals involved often have prior close affiliations, and that the derived distinction between cooperation and coordination dilemmas can become blurred (Henrich & Muthukrishna, 2021; Ostrom, 1990; Wilson, 2015; Wilson et al., 2013). These differences between experimental settings lead to questions about the transferability of experimental results, such as: do the rates of reciprocity observed in the lab translate to real work contexts, and are behavioural types actually observable in natural dilemmas or are they merely artefacts of experimental contexts?

The move to substantiate the experimental findings on reciprocity with naturalistic data is promising. Anthropological studies of hunter-gatherer societies have found observational evidence of reciprocity and cooperation in behaviours such as food sharing (J. M. Koster & Leckie, 2014), and there are examples of inter-organizational cooperation in market economies, including firm collusion (Asker & Nocke, 2021). For example, Mao and Song (2021) observed that reciprocity plays a key role in analysts’ propensity to underwrite securities between firms. Furthermore, Frey (2017, 2019) measured reciprocity in an online video game and detected comparable behavioural types to those found in experiments, although this is still an emulation of a real-world environment.

A limiting factor of the study of naturalistic cooperation has been a paucity of behavioural data that parallel experimental settings. Here, we analyse a novel, granular dataset on shared bulk-purchasing interactions that are similar to some experimental public goods settings to determine how reciprocity and behavioural types emerge in a real-world setting. The data consist of individuals extending mutual aid to ensure that their needs and wants are met and that the club continues to function. We identify economic reciprocity at degrees that exceed most experimental studies, and we derive novel behavioural types that tend towards a stable reciprocal strategy.

## 2. Food clubs

Food-buying clubs are semi-formal organizations that collectively arrange wholesale food purchases from national and regional distributors (Hupper, 2019; Lange, 2022; Tremblay, 2017). Clubs range in size from 5–100 members, and consist of one or two coordinators that maintain distributor accounts, and general members who only participate in purchasing. Orders are placed on a weekly or monthly basis, and items range from individual or family-sized goods to bulk items with more supply than one buyer generally needs.

These clubs constitute miniature, informal consumer co-operatives,^1^ as they are formed to take advantage of the discounts afforded by wholesale bulk purchasing and gain access to specialty goods that may be unavailable in local stores (Little et al., 2010). Food from local producers and niche items such as those labelled organic or fair trade are not always supplied by the traditional food system, particularly in rural communities, because demand has not reached a profitable scale for large chain grocers (Deller et al., 2017; Little et al., 2010). However, supply can be provided by a member-owned co-operative grocery store, where motivations are not exclusively profit driven and members collectively fulfil the missing market for the demand that does exist (Deller et al., 2017; Steinman, 2020). In the absence of a formal co-operative store, food-buying clubs offer a similar opportunity for motivated individuals, as well as for those who wish to begin the process of forming a co-operative grocery store (Hupper, 2019; Lange, 2022; Little et al., 2010; Tremblay & Waring, 2015).

As co-operative organizations, food-buying clubs rely on cooperation much more than a typical hierarchical organization. Co-operatives are defined by their democratic decision-making, member ownership, and reliance on cooperation (Birchall, 1997), and members of co-operatives tend to identify trust and reciprocity as integral to co-operative success (Pesämaa et al., 2013). Members of co-operatives have been found to exhibit higher levels of behavioural cooperation as elicited by the dictator game (Tremblay et al., 2019), but it is unclear whether and how that cooperation is maintained. Furthermore, while formal co-operative grocery stores can potentially rely on established rules to avert or solve social dilemmas (Waring et al., 2022), food-buying clubs often lack institutions initially, making reciprocity essential for early club success (Hupper, 2019).

Clubs facilitate supply by pooling their demand to buy goods in bulk, e.g. flats of cans or 25–50lb bags of dry goods like flour. In most instances, the demand of a single buyer is less than the minimum amount required by distributors, or ‘case size’, so multiple members are required to buy in and ‘co-purchase’ the item. For a co-purchase to be successful, members must combine their buy-ins to match the case size, and doing so requires a negotiation of their preferences for all available goods. This dynamic can lead to a social dilemma such that if preferred amounts don’t match case sizes exactly, members need to decide who will buy more or less so that the case is filled.

Previous research of these clubs has shown that club members are exceptionally cooperative in behavioural games. Members’ propensity for cooperation has been measured using a dictator game (DG) and a public goods game (PGG), in both of which club members contributed unusually large fractions of their endowment (DG: 58%, PGG: 71%) (Hupper, 2019). These contribution levels substantially exceed those reported in the literature for equivalent games, as the next-largest mean contributions are DG: 48% in Paciotti et al. (2011), and PGG: 57% in Apicella et al. (2012) and 58% in Henrich et al. (2001). Qualitative responses of our sample reported in Hupper (2019) corroborate an abundance of cooperation among active clubs, and find that members experience stress when cooperation is not sufficient. Coordinators of some clubs stated ‘Members need to contribute more’, while others said ‘For the most part members carry their weight’, or ‘Everybody steps up.’

### 2.1. The co-purchasing dilemma

The relationship between what members want and what they end up buying can be described as a game. Imagine a co-purchasable item as a game played between a subset *m* of the club’s *M* members (*m* ∈ *M*). The item is a case of *X* single shares, and each share costs price *p*. Shares can be aggregated into quantities of *x* shares (*x* ≤ *X*) with an aggregate price of *x* * *p*. We imagine that members might only prefer to buy a specific number of *x* shares for a given item, and otherwise prefer to keep their money for other share quantities. To illustrate, we define members’ ‘utility’, or hedonic value (Fehr & Rangel, 2011), as a function of *x* and their exogenous level of money, or ‘wealth’ *W*:
(1)
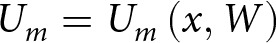


At *x* = 0 (no shares purchased), members have a base level of utility endowed by just their wealth, *U_m_^base^*. As shares *x* increase, members pay *p* * *x* from their wealth, and utility deviates from *U_m_^base^*. This trade-off allows us to formally define when members have a preference for the item: members prefer the item at share quantity *x* IFF the utility at that quantity is greater than or equal to their base level of utility:
(2)
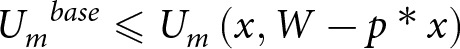


If individuals only make a purchase when they have a preference for the item, we can define the purchase decision for an individual at each subset quantity, *C_xm_*, as
(3)
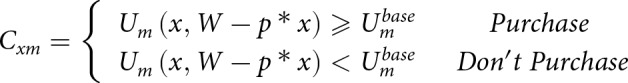


**Figure 1.**
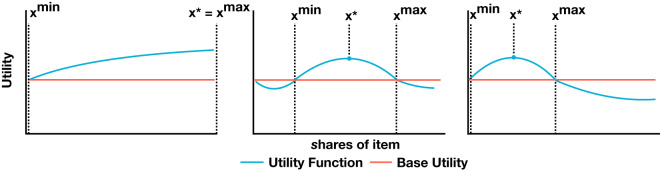
**Three hypothetical member utility functions (blue) compared to the base utility (red) across all share** x **quantities.** Left: The member utility strictly increases from base utility with decreasing marginal returns. As such, x* = x^max^ = X. Center: Utility is less than the base at low and high share quantities but exceeds it at medium quantities with an optimum at x*. Right: Utility exceeds base utility at smaller share quantities with an optimum at x*, but is less at medium and higher quantities.

This utility curve has many potential shapes (Fig. 1). As previous studies of food clubs have observed members occasionally purchasing entire co-purchasable items individually (Hupper, 2019; Lange, 2022), some members may have utility functions like that in the left-hand panel of Fig. 1 for some goods. However, these instances are rare. We expect most utility functions to look like the middle and right-hand panels of Fig. 1, where there is some range from *x^min^* to *x^max^* that satisfies the purchase condition of Equation 3, with some optimal quantity x* endowing the most utility. Not pictured is the final possible shape, where there is no subquantity that satisfies the purchase condition, i.e. *x^min^* = x* = *x^max^* = 0, indicating that the individual has no preference for the item.

Comparing possible aggregations of member demand and its relation to *X*, i.e. how members arrive at the case size, allows us to theoretically define behaviours across the prosociality spectrum. If all members are able to meet the case size exactly with their optimal share quantity, i.e. 
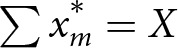
, the co-purchase succeeds with purely self-interested coordination, as every individual will receive their optimal share. However, if members’ optimal share quantities do not meet the case size, i.e. 
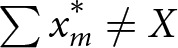
, some or all participating members will need to change their share and accept some utility cost in order for the co-purchase to succeed. This requires cooperation, or altruism.

The costs incurred by a member in completing a shared purchase defines their action as cooperation or altruism. When a member’s purchase quantity deviates from x*, but lies between *x_m_^min^* and *x_m_^max^*, it is an act of cooperation. In this case, the member still receives more utility than they would with an equivalent amount of money, but it is less than they would have if they could purchase their preferred x* shares. Going further, if a member needs to accept a quantity that is outside of the preferred purchase range, i.e. does not satisfy Equation 3, doing so would require some degree of altruism. The utility cost is the difference between *x_m_* and x_m_* and the added cost of forgoing the equivalent amount of money.

This simple structure shows how free riding works in food clubs. For a single co-purchased item to succeed, members solicit others to accept a utility cost by purchasing more or less than they would prefer. The members who assist in this way are acting cooperatively and sometimes altruistically. Therefore, the co-purchasing game is a variable social dilemma that depends on members’ preferences and item factors such as case size. Cooperators or altruists must hope to recoup their utility losses through reciprocity on other items.

**Figure 2.**
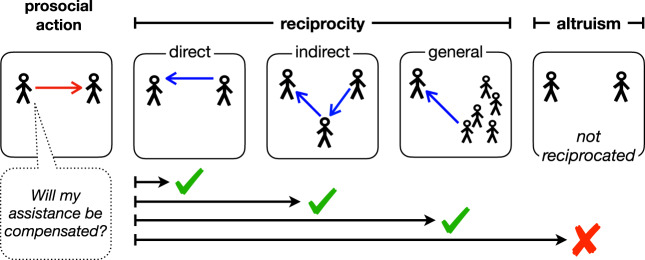
**Reciprocity determines behavioural classification after the fact.** Cooperative assistance in food-buying clubs is only beneficial to the cooperator if it is reciprocated. Reciprocation may occur at greater lengths of time and social distance, and be either direct (dyadic, often short term), indirect (cyclic, often medium term), or general (from the group, possibly long term). Evidence of cooperation derives from greater occurrence of direct and indirect reciprocity.

In many real-world scenarios, it may not be possible to define an action as cooperative or altruistic at a given moment in time, even from the perspective of the actor (Wilson, 2015), and co-purchasing is no different (Fig. 2). Individuals might be expecting a cooperative act to be reciprocated, and the act may not have been intended as altruistic. If it is reciprocated, the individual may think of it as successful cooperation through reciprocity. If it is never reciprocated, the individual may come to consider it as an altruistic outlay. Club members may not know how to view a contribution to a co-purchasing effort until long afterwards.

### 2.2. Comparable theoretical dilemmas

Because co-purchases are rivalrous (i.e. members’ shares cannot also be purchased by other members) but non-excludable (i.e. any member can potentially purchase a share), they are common pool resources by definition (Reiss, 2021). However, traditional common pool resource games (e.g. Bru et al., 2003) usually require participants to choose an amount to take from a resource without having to contribute to it. This does not adequately reflect the decisions made in co-purchases, as individuals are pledging an amount of money to exchange for the provision of the good. Furthermore, while there are thresholds in common pool resource games, meeting them results in the collapse and non-provision of the resource, while thresholds *must* be met for co-purchased items to be provided.

An economic game that more closely emulates the contribution requirements and threshold conditions of co-purchases is the threshold public good game with refund (TPGR) (Cadsby & Maynes, 1999; Cartwright & Stepanova, 2015). TPGR games require individuals to pledge endowed money to a common fund where it will be multiplied and redistributed equally if the common fund reaches a required threshold, but pledged money is returned to recipients if the threshold is not met (Cartwright & Stepanova, 2015). The case sizes of each co-purchase are an example of what thresholds emulate, as not meeting them results in the item’s not being ordered and the return of money previously pledged.

Co-purchases deviate from the TPGR model in that the goods provided are a unit price reduction and personal utility, and multiple interactions are dispersed across a network where purchasing partners can change. Successfully reaching the threshold in TPGR game results in a uniformly divided monetary payout of the common fund plus a growth factor chosen by the researcher (Cadsby & Maynes, 1999; Cartwright & Stepanova, 2015). Meanwhile, while the unit price decrease of a successful co-purchasing dilemma could be considered a ‘payout’, total savings relative to buying the good elsewhere still differ according to members’ relative pledges. Additionally, accurately measuring the full hedonic utility payout of receiving the good (which is the most important because it drives demand) would require additional data beyond what was collected for this study (Fehr & Rangel, 2011). Furthermore, while individuals do receive utility from money they would have spent on the good if a co-purchase is unsuccessful, those whose required *x^m^* would have satisfied the purchase condition in Equation 3 incur a utility cost by the item’s not being purchased, whereas TPGR games refunds have no such cost. Finally, traditional TPGR games generally assume sustained interactions in each game, while the member composition of co-purchasing interactions can vary across items and orders, with the potential for old members leaving the club and new members entering for every order.

While these deviations are distinct, and a full formal model of the co-purchasing dilemma is yet to be built, the procedural likeness of co-purchases to TPGR games provides a novel opportunity to compare a nuanced real-world scenario to straightforward theoretical predictions. First, co-purchasing represents a clear collective action dilemma with real monetary and utility trade-offs that are vulnerable to free riding, which can be difficult to capture data on in naturalistic settings. Second, they often lack formal institutions to reinforce cooperation by lowering its cost or penalizing free riding, making them prime subjects to study how prosociality is maintained through reciprocity (or not) early in the organizational lifespan. While a full analysis of the life cycle of these clubs is beyond the scope of this study, establishing an understanding of how reciprocity unfolds will generate insights into future studies of that nature. Finally, the data we have on these clubs allow for the granular measurement of prosocial behaviours that club members acknowledge occurs and is theoretically important to early organizational success. As such, we are able to complete a novel assessment of real-world prosociality that lays the groundwork for future naturalistic study.

## 3. Methods

### 3.1. Dataset

Our dataset consists of a set of purchasing records from two different software platforms used by food-buying clubs to coordinate purchases. Platform 1 provided data for 30 clubs from late 2011 to early 2017, and platform 2 provided data for 19 clubs from early 2010 to early 2019, for a total of 107 club years. The clubs in our dataset are samples from WEIRD countries: 2 from Australia, 1 from Canada, and the rest from the USA. For our analysis, we removed all purchases of individual items as well as clubs with no bulk purchases. The final combined dataset contained 35 clubs, with 1,528 individuals across 1,341 orders, for a total of 10,261 co-purchases.

Data from our providers only included software usernames and purchasing details, but no personal information. As such we were unable to ascertain differences in reciprocity or cooperation by age or gender identity. Furthermore, our sample includes both defunct and still-functioning clubs (as of the end of the data period), though the effect of reciprocity and cooperation on the functional status of these clubs is beyond the scope of this paper and is not a part of our analyses. See supplementary materials for club-level summary statistics.

### 3.2. Co-purchasing network construction

**Figure 3.**
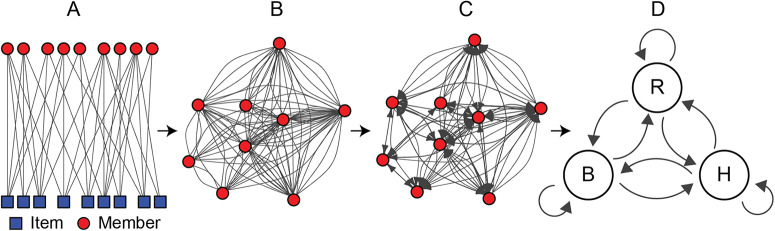
**Co-purchasing networks are projected to count reciprocity and ascertaining member types.** Purchase data are used to create a (A) bipartite network between members (red circles) and bulk food items (blue squares), which is projected as a (B) co-purchasing network between members that purchased the same items(s). Edge directionality is assigned according to an individual’s relative share of the bulk item(s), to produce a (C) directed co-purchasing network, from which (D) individual Markov transition probabilities between behaviours are derived.

We used a network-based method to study reciprocity between members over time (Fig. 3). To detect reciprocity in co-purchases, we construct a bipartite purchasing network for each order by connecting members to the items that they purchased (all networks were generated and analysed using R (Bates et al., 2021; R Core Team, 2022; Csárdi et al., 2023; Song et al., 2019; Wickham et al., 2017, 2018)). We then use this to create a unipartite co-purchasing network where members are connected to each other by their co-purchases, ensuring that multiple co-purchased items result in multiple edges per pair of members. Edge directionality is then assigned according to the ordinal volume of co-purchase share. Arrows point to the member who purchased more in each dyad and are split into two directed edges pointing at both members when equal shares are purchased. We do this by building off the assumption that share size is directly proportional to members’ relative utility, i.e. individuals who purchase more tend to derive more utility from the item, and stand to lose more utility if the bulk purchase is not completed. As such, members with lesser shares are assisting those who need to purchase more to derive their optimal utility. This method is corroborated by a negative correlation between the order in which members pledge to purchase shares of a bulk purchase and the amount they buy (Spearman’s *ρ* = − .65, p *<* .001; data only available from one of the software platforms). In other words, individuals who join a bulk purchase later tend to buy smaller shares. We term this “share-based” assistance, and it allows us to delineate multiple levels of economic reciprocity.

### 3.3. Identifying reciprocity

We organize patterns of reciprocity into separate categories based upon social and temporal proximity. We define the reciprocity of a club member, *m*, to others in the club by the relationship between their total in-degree, *k*^in^*_m_*, (number of times they were helped) and total out-degree *k*^out^*_m_* (number of times they have helped). For each order, we denote the outgoing edges of *m* as reciprocal if they have a matching in-edge. For example, suppose two members, *m*_1_ and *m*_2_, co-purchase shares of two items, A and B. If *m*_1_ purchases a larger share of A than *m*_2_, and *m*_2_ purchases a larger share of B than *m*_1_, we consider these co-purchases to be reciprocal in terms of relative share. Doing so allows us to categorize edges as either reciprocated or unreciprocated and count the number of each across all orders.

We differentiate reciprocity as direct and indirect, and separate reciprocal behaviour within the same order and between different orders. When calculating the different categories of reciprocity, we assume that social and temporal closeness are salient to economic transactions and should be respected when interpreting empirical patterns. This is in line with prior research findings that direct reciprocity is more consequential in sustaining cooperation when indirect reciprocity is also present (Roberts, 2008; Schmid et al., 2021), and is logically consistent. We therefore count direct reciprocity before indirect and reciprocity within orders before doing so across orders. See Table 1 and supporting text our supplemental materials for a full explanation of the accounting method.

Finally, we estimate the global average rate of reciprocity per individual for comparison with laboratory studies. Previous studies have measured reciprocity by regressing subjects’ donations on group-level average donations with subject-level fixed or random effects (Croson et al., 2005; Smith et al., 2018). To compare reciprocity in food buying clubs with the results of previous studies, we specify a generalized linear model (GLM) of individuals’ total out-degrees as a function of total in-degrees and group-level random effects:
(4)
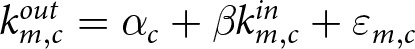


In (4), *α_c_* is a random intercept term that allows us to account for club-level variation such as unobserved socio-demographics, and *ε_mc_* is an error term. The *β* parameter may be interpreted as the global average percentage of in-degrees that are reciprocated across all individuals in all clubs in our sample, or the average number of out-degrees extended per in-degree. As such, it is comparable to coefficients from other studies that model average public goods donations as a function average group donation (Croson et al., 2005; Smith et al., 2018).

### 3.4. Identifying cooperation and altruism

Because the clubs in our study are small and bulk purchases are common, we expect to see some degree of reciprocity due to rational coordination; variation in preference overlap inevitably results in members buying different share sizes of many items to others in the club. We also know that club members acknowledge that cooperation is often involved in shared purchasing, and members report dissatisfaction and inequality in purchasing assistance (Hupper, 2017; Lange, 2022). We therefore investigate the strength of reciprocity as it reinforces prosociality across the spectrum, and how this varies across clubs. Significant variation in the extent of reciprocity would suggest that these clubs are solving their social dilemma in different ways, or not at all, consistent with the conditionality of prosocial behaviour.

To improve inference regarding the prevalence of altruism, we use a restrictive set of criteria to identify the economic interactions that are most likely to be altruistic. The co-purchases that are most likely driven by altruism are those in which one member helps another member to purchase an item which is outside of their revealed preference set (here defined as having purchased an item at least twice). To do so, we look for interactions we term ‘singular assistance’, where individuals contribute only once to an item that has been purchased multiple times. To illustrate, suppose a member, *m*_1_, purchases shares of a bulk item in different orders, and member *m*_2_ never buys that item across any orders except for a single interaction in which they bought a smaller portion of the item than *m*_1_. This purchasing pattern may suggest that *m*_1_ prefers the item and *m*_2_ does not, even after purchasing some of it in one round, so it is most plausible to conclude that *m*_2_ was altruistically (or at least cooperatively) assisting *m*_1_. Reciprocity of singular assistance would then occur if *m*_1_ assists *m*_2_ in the same way in a later order. We estimate the regression model given by Equation 4 using members’ singular assistance degrees to assess global average singular assistance reciprocity.

### 3.5. Stability of reciprocity

Counting of types of reciprocal edges in each order also allows us to compute their proportions over time. With this information we can look for variability in the patterns of reciprocity within groups as well as measure their general temporal stability. Since the time between orders varies within and between clubs, a traditional time-series stability analysis such as an augmented Dickey–Fuller (Dickey & Fuller, 1981) or Philips–Perron (Phillips & Perron, 1988) test would produce biased estimates of stability. Instead, we quantify stability as the degree of temporal variation in each reciprocity category represented by its coefficient of variation, *c_v_*, as a simple measure of variability. Smaller *c_v_* values indicate greater temporal stability and vice versa.

### 3.6. Behavioural types

We use individuals’ behaviour across all orders to categorize them into different behavioural types, analogous to the general behavioural types identified in laboratory circumstances (Andreozzi et al., 2020; Fischbacher et al., 2001; Frey, 2017). To do so, we calculate members’ shared purchase ratio (SPR), which is the log ratio of their order level out- and in-degrees. For club member *m* in order *t*:
(5)
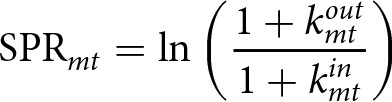


Taking the logarithm transforms the ratio into an easily interpretable magnitude: a positive SPR indicates that an individual gave more assistance than they received, a negative SPR indicates they received more than they gave, and a SPR of zero indicates perfect reciprocity. As individuals could give almost as much assistance as they have received (or vice versa) and still be considered reciprocal by their peers (Molm, 2010; Trivers, 1971), we use a clustering approach to allow for data-driven flexibility in classification. For each club, members’ SPRs are pooled across all orders and divided into three types using univariate k-means clustering with three means designated a priori:

- **Helpers:** designated cluster centred at less than zero, i.e. *k*^out^*_mt_* *>* *k*^in^*_mt_*
- **Reciprocators**: cluster centred at zero, i.e. *k*^out^*_mt_* = *k*^in^*_mt_*
- **Beneficiaries**: cluster centred above zero, i.e. *k*^out^*_mt_* < *k*^in^*_mt_*

K-means clustering algorithms traditionally optimize all cluster centres (Steinley, 2006), but we modify the algorithm to fix the reciprocator centre at zero, i.e., perfect reciprocity. If a SPR falls equidistant between the reciprocator centre and either of the other centres, we assign it to either the beneficiary or helper cluster to maintain conservative estimates.

To determine members’ dominant behavioural type, we analyse type changes through time by calculating transition probabilities using a Markov chain (Spedicato et al., 2021). Markov chains are used to model systems where transitions occur between different states and assume that the next state is exclusively dependent on the current state (Norris, 1997). While a Markov chain with memory would provide more precise predictions about members’ evolution over time, traditional Markov chains produce good approximations of the stationary distributions of systems with memory, especially in systems with limited states such as ours (Wu & Chu, 2017). As such, we assign each member a dominant type based on which has the largest probability in the stationary distribution of their transition matrix. We report the dominant type assignments for individuals in each club, as well as a global-level average transition matrix (part D in Figure 3), which gives us a measurement of the stability of each role across clubs and which is the predominant behavioural type in each order (see supplemental materials Table 5 for club specific cluster centres).

### 3.7. Robustness check

While accounting for assistance using relative share size is consistent with standard utility theory, it is possible that it is not always appropriate. For example, club members may reciprocate the responsibility for initiating the purchase process of mutually desired goods. Furthermore, the timing of a purchase may be another indicator of the utility endowed by an item, i.e. individuals purchase into items they want sooner in the order process than for those who want less.

To account for this possibility, we checked the robustness of our results with the 13 clubs whose software provider gave us information on the timing of member purchasing. We reassigned the directionality from Fig. 2C for co-purchasing networks based on *when* members commit to purchasing an item. For this purchase order definition of assistance, members who purchase items later are assumed to be aiding those purchase sooner. From these new networks, we re-count each type of reciprocity and re-estimate our GLM.

## 4. Results

We find that club members are highly reciprocal in their purchasing patterns overall. The majority (60%) of reciprocity occurs directy and within orders. We also find that reciprocators are the most common and the most stable behavioural type.

### 4.1. Reciprocity types

In classifying different types of reciprocity, we observe within-order direct reciprocity as the most frequent form, with 60% of the average club’s edges classified as directly reciprocal within orders (Fig. 3A). Directly reciprocal between-order edges represent a far second, followed by indirectly reciprocal edges within orders and between orders (see supplemental materials Table 2 for percentages of each). Finally, 17% of all edges remained unreciprocated as of the final observed order.

Globally, our GLM indicates that members reciprocate 88% of the co-purchasing assistance they receive (*β* = .88, p *<* 0.001). This can be considered an upper bound reciprocity that covers the whole suite of prosocial behaviours, including altruism, cooperation, and coordination. Using the more restrictive singular assistance measure we find that individuals reciprocate 46% of the co-purchasing help they receive on average (*β* = .46, p *<* 0.001). As this metric most closely measures reciprocated altruistic cooperation, this can be considered the lower bound estimate of reciprocity. See supplemental materials Table 4 for full model results.

### 4.2. Temporal variation

We find that the variability of each type of reciprocity corresponds inversely to the pattern of prevalence (Fig. 3B). For example, the proportion of directly reciprocal edges within orders have the least temporal variation (and are therefore the most stable), both temporal forms of direct reciprocity vary less over time than indirect reciprocity, and within-order reciprocity is less variable than reciprocity between orders.

### 4.3. Behavioural types

**Figure 4.**
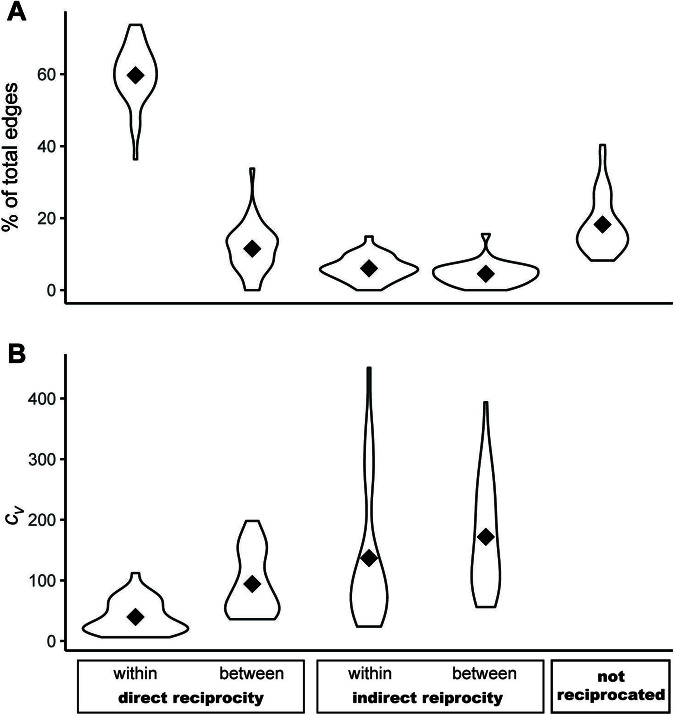
**Rapid, direct reciprocity is most common and most stable.** (A) More than half of all bulk purchasing is reciprocated directly and rapidly (within the same order). Diamonds represent mean club abundance of each reciprocity type. (B) Within-order, direct reciprocity is also the most stable. Diamonds represent mean coefficient of variation, *c_v_*, by club.

**Figure 5.**
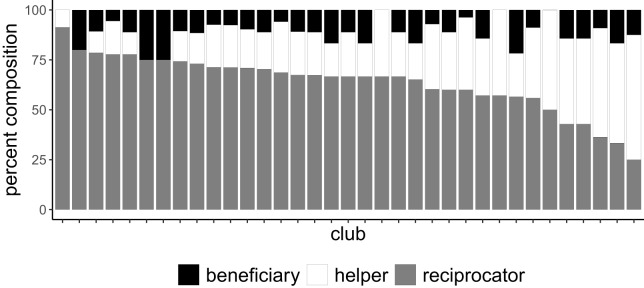
**Reciprocators are the most abundant member type.** Club composition by member type, arranged in descending order of proportion of reciprocators. Reciprocators are the most abundant type, followed by helpers and beneficiaries, who remain consistently below 25% of members.

**Figure 6.**
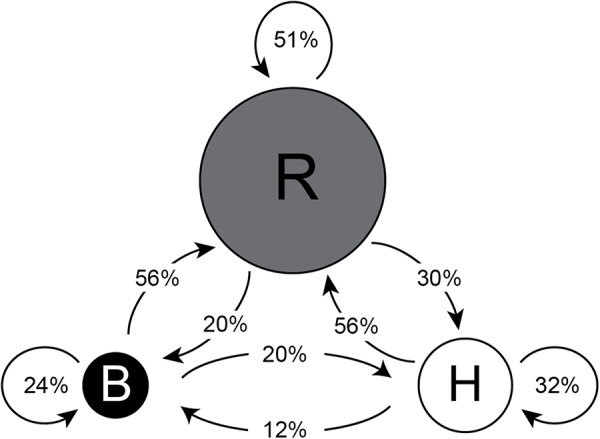
**Reciprocator is the most stable behavioural type within orders.** Reciprocator type is the average absorbing state, with a greater than 50% chance for each role to be a reciprocator in the next order, globally. Circle size is proportional to the sum of all incoming transition probabilities.

66.1% of club members were classified as dominant reciprocators by the stationary distribution of their Markov chains. Across all clubs, reciprocators are the most abundant member type, with an average proportion of 65.3%, followed by helpers (29.4%) and beneficiaries (12.4%) (Fig. 4). Reciprocator is also the absorbing state of the global average transition matrix, where the probability for staying a reciprocator in the next order is 51%, and the probabilities for becoming a reciprocator from helper and beneficiary are 55.7% and 56.2% respectively. Fig. 5 shows the member type composition of each club in a stacked bar graph, and Fig. 6 shows the global average transition probabilities. See supplemental materials in Table 5 for SPR cluster centres, and Table 6 for member type counts.

### 4.4. Robustness check

**Figure 7.**
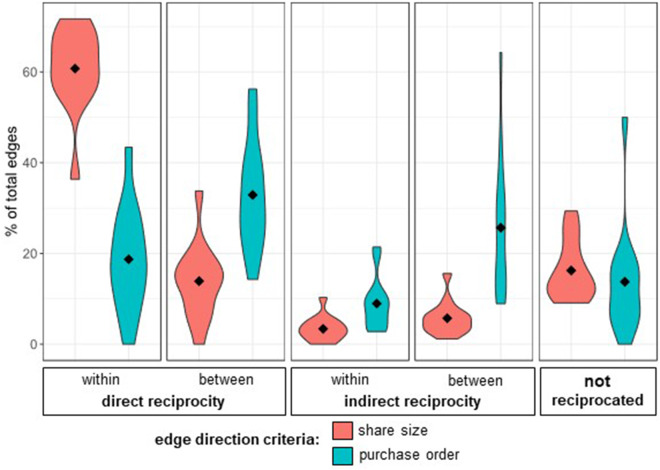
**Changing the criteria of assistance induces changes in the average timing of reciprocity.** By defining assistance by purchase order, there is a substantial increase in the amount of reciprocity occurring between orders, directly and indirectly. There is also a large decrease in within-order direct reciprocity. Most bulk purchasing is reciprocated directly (by the beneficiary) and over time (between orders). Global means are shown as black diamonds.

Defining assistance by purchase order for a subset of clubs induces changes in the patterns of observed reciprocity (Figure 7). Our random effects estimation of the altered networks estimates that individuals reciprocate approximately 63% of the assistance they are given (*β* = .63, p *<* 0.001). We also find that edges classified as directly reciprocal *between* orders are most abundant with 32.9%, followed by indirectly reciprocated between orders (25.7%), and within-order direct (18.7%) and indirect (8.9%).

## 5. Discussion

The observed patterns of economic cooperation in small food clubs provide some of the first estimates of reciprocity rates and the emergence of behavioural types in a real-world organizational scenario. The degree of economic reciprocity exhibited in these clubs is, to our knowledge, unprecedented in the empirical literature, and it supports behaviour across the prosociality spectrum. Our findings are supported by multiple lines of evidence, including a high mean level of reciprocity across clubs, the existence of multiple types of reciprocity (direct and indirect, short-term and longer-term), the predominance of the reciprocator behavioural type within clubs, and a robustness check.

*High levels of reciprocity.* Our GLM estimated that 88% of all edges are reciprocated, as are 46% of singular assistance edges. The all-edge model represents a ceiling value that combines interactions ranging from coordination to altruism. The singular assistance model used a highly restrictive definition of cooperative edges to approximate the floor value that would be indicative of just cooperation and altruism, as individuals would probably have bought the co-purchased items multiple times if they had borne any utility.

We find evidence that we are measuring the full spectrum of prosociality by comparing our model estimates with the average donations of economic games played with these groups in previous research (Hupper, 2019). The results of our total edge model correspond with the results of a PGG (*β* = .88, PGG mean donation = 71%) and the singular assistance results correspond with the results of the DG (*β* = .46, DG mean donation = 58%). Dictator games elicit the propensity for unenforced fairness and altruism towards another individual (Engel, 2011), and our singular assistance edges were restricted to identify similar behaviour in a real-world context, as they can only link to a single person, the purchase initiator. Concurrently, public goods games measure how cooperative individuals are towards an entire group (Zelmer, 2003), and our total edge model captured the reciprocity directed at multiple members of co-purchases. This apparent alignment between parameters and game donation amounts may imply that our singular assistance model measures actual reciprocal altruism, while our total edge model represents individuals being prosocial in general to support the club. However, the inevitable presence of coordination-based reciprocity in our total edge model makes absolute comparison imperfect.

Further evidence of our claim comes in the comparison of our estimates to previous studies of conditional cooperation that compare individuals’ donations in PGGs to the mean group donation or subjects’ perception thereof. As club members’ in-degrees indicate the total amount of assistance given to them from their clubmates, they are tantamount to subjects’ payouts in experimental PGGs and are a measure of average group-level donation as experienced by the club member. Members’ out-degrees are consequently the amount of assistance they have given and are comparable to donations in experiments. In all cases, our total edge model exceeds previous estimates of conditional cooperation, and our single assistance model falls within their range.

In anonymous PGGs, Croson et al. (2005) found that people gave an average of 40% of the observed group donation (Table 1: Voluntary contribution mechanism in Croson et al., 2005). In another anonymous PGG, Fischbacher and Gächter (2010) found that individuals gave 67% of what they believed the rest of the group gave (Table 2: Model 3 in Fischbacher and Gächter, 2010). Using an instrumental variable approach in an anonymous PGG, Smith (2013) found that individuals gave between 45 and 57% of what they believed others had given in the previous round (Table 2 in Smith, 2013), depending on model specification. Finally, in PGGs with Hazda hunter-gatherers, Smith et al. (2018) found that people donated 55% of the average group donation (Table S4, Model 1 of Smith et al. 2018). All of these studies utilize a linear public goods set-up, which is specifically designed to elicit behaviour that incurs a personal cost, i.e. cooperation and altruism. Since our singular assistance reciprocity estimate falls well within these studies, we believe we are likely observing reciprocal altruism, or cooperation at the very least. Furthermore, the outlay of our total edge model from previous studies suggests that we are observing reciprocity that supports the full spectrum of prosocial behaviours, including coordination.

One possible explanation for the our observed rates of reciprocity is that our groups hail from WEIRD, or Western, educated, industrialized, rich, and democratic countries (Henrich, 2020). WEIRD societies have greater degrees of market integration, which has been theorized to increase interpersonal cooperation (Henrich, 2020). Cross-cultural studies that compare group average cooperation in economic games and various measures of social development and market integration have been employed to test this hypothesis (Henrich et al., 2001). For example, Henrich et al. (2010) found that individuals from countries with more market integration tend to exhibit higher levels of cooperation in economic games. Furthermore, individuals from firm-type organizations within WEIRD societies, including managers and general employees, perceive that prosociality is fundamental to organizational success (F. Koster & Sanders, 2006). Since our clubs are *facilitating* market integration by supplying a missing market, higher prosociality may be required.

*Predominance of direct reciprocity*. Direct reciprocity is generally more effective at supporting cooperation than indirect reciprocity, and does so to a greater degree when they are allowed to co-evolve (Roberts, 2008; Schmid et al., 2021). When assessing directional assistance by share size, our findings support this assertion, as we found that most reciprocity occurred between pairs of individuals and on a short timescale (about 1–4 weeks). 72% of all reciprocal interactions and 60% of all co-purchasing was attributable to within-order direct reciprocity. Though assessing directional assistance through purchase orders in our robustness check decreases the amount of direct reciprocity within orders, direct reciprocity is still most prevalent. This suggests that co-purchasing reciprocity supports prosocial co-purchasing behaviour, at least in the short term, and that club members resolve this as immediately and directly as possible. If buying clubs did not require prosociality – and cooperation or altruism specifically – between members, we would expect less reciprocity of all types, and especially less direct reciprocity.

Our results also show how dyadic reciprocal interactions can undergird the cooperative success of group-level social dilemmas. This parallels research in which experimental subjects alternated between PGGs and dyadic prisoner’s dilemmas. A study by Rand et al. (2009) found that cooperators who were rewarded with cooperation (and free riders who were punished via defection) in the prisoner’s dilemma rounds tended to cooperate more in the public goods rounds. This same dynamic could be unfolding in the buying clubs we study, as members who aid in buying larger items that require extra individuals to contribute could be rewarded with assistance on smaller items that they initiate, or could be punished for not doing so by having their items go unfulfilled. However, testing such an assertion would require an analysis that is beyond the scope of the current work.

More socially and temporally distant forms of reciprocity are also detected when assessing assistance by share. Theory and simulation reveal that direct and indirect reciprocity evolve simultaneously to support cooperation, with direct reciprocity playing a more prominent role (Boyd & Richerson, 1989; Nowak & Sigmund, 2005; Roberts, 2008), and our results suggest that our sample corroborates this theory. If only direct reciprocity were at play, we would only see co-purchases where many individuals purchase the same amount of an item, or individuals trading off on who buys the lion’s share of an item. Similarly, if only indirect reciprocity were at play, we might mostly find instances where individuals buy the largest portion of one item with one person and the smallest portion of another item with someone else. Since we detect combinations of both (among other reciprocal patterns), our results suggest that the theoretical predictions of how direct and indirect reciprocity reinforce cooperation play out in this naturalistic setting.

The differences in reciprocity patterns when using a purchase order-based system may reveal more about clubs’ purchase processes. To begin, the average global reciprocity rate is 25 percentage points lower than the share-based criterion. This could be due to arbitrary circumstances that delay individuals’ ability to pledge, such as delays to their ability to log into the software. As the utility assumptions are consistent across both criteria (individuals who want or need an item more should hypothetically buy more of it and do so sooner), these reasons could also explain why the correlation between share size and purchase order isn’t stronger. Additionally, the increase in between-order reciprocity could indicate that individuals are trading off responsibility for initiating purchases. If individuals alternate initiating items each order, we would expect to find more between-order reciprocity rather than within order, as we do. Despite the change in temporal order, we still find that direct reciprocity is more abundant than indirect, which is further evidence that direct reciprocity plays a key role in successful co-purchasing.

*Behavioural types*. In the initial work of Fischbacher et al. (2001), individuals were classified based on the proportion of their endowment that they gave: free riders kept all or most of their endowment, altruists gave all or most of their endowment, and conditional cooperators gave around the group average from the previous round. While intuitive, using these classifications to describe real-world behaviour is of limited use because experiments oversimplify the dynamics of naturalistic settings. Studies of strategies and behaviours in the real world further highlight that complexity rarely allows for straightforward behavioural classification (Efferson et al., 2023; Mesoudi et al., 2016), leading to the possibility that behavioural types are experimental contrivances.

While there is a logical correspondence between the behaviours exhibited in our groups and those found in experiments, the differences are notable in definition and emergence. Conditional cooperators and reciprocators have the most equivalency, as conditional cooperators do what the group does on average and reciprocators are generally giving what has been given to them. Free riders in experiments line up with beneficiaries, as both gain more than they give, and helpers line up with altruists for the opposite reason. However, despite having a usual type, many members still alternate from order to order, making their behaviour less straightforward than would be expected from experiments. Specifically, beneficiaries still render assistance (albeit less often), making their behaviour less opportunistic in absolute terms than that of traditional free riders. Similarly, helpers still benefit from assistance in most orders, indicating that their role is less self-sacrificial than that of their altruistic counterparts in experiments.

This leads to the conclusion that in naturalistic settings such as food clubs, cooperation and reciprocity appear as probabilistic patterns rather than definitive classifications, at least in the case of our study. Experiments have analysed the stability of behavioural types across time by retesting previous subjects and found that individuals tend to repeat their behaviour (Andreozzi et al., 2020; Kocher et al., 2008). While we also observe repeat behaviours, we more often observe members fluctuating between the roles, with a tendency to default to one more often than the others. Furthermore, reciprocity appears to be the most stable strategy of them all, as more individuals across all clubs tend to default to this role, and it acts as the absorbing state in the global transition matrix.

As conditional cooperators and reciprocators have the most in common, it is useful to compare our observed proportion of reciprocators with those of conditional cooperators in the experimental literature. The percentage of individuals who were classified as consistent reciprocators was 68.5%, which is one of the highest proportions found in the literature to date. Fischbacher et al. (2001) were the first to identify behavioural types by correlating subjects’ donations with the average donation of the group with the focal individual’s donation removed, and classified 50% of their subjects as conditional cooperators. In a review of 17 replications of Fischbacher et al. (2001), Thöni and Volk (2018) found proportions of conditional cooperators ranging from 40 to 77%, with an average of 62%; of the studies reviewed, only 2 studies exceeded our proportion. In an organizational setting, Deversi et al. (2020) found approximately 41% conditional cooperators (called ‘matchers’ in the text) in a company-wide experiment using a clustering classification method.

Finally, Frey (2017) found that 39% of individuals playing an online video game with a threshold public goods mechanism could be classified as conditional cooperators by correlating the individual’s effort with the efforts of others in the game.

These comparisons indicate that members of our clubs are more likely than most individuals in public goods experiments to be reciprocators/conditional cooperators, and that conditional cooperation and reciprocity may be higher in naturalistic public goods situations among consistent peers with pre-established relationships, or where individuals interact outside of the collective action dilemma. Furthermore, we may observe more reciprocators because cooperation and coordination are intermixed in these dilemmas. Experimental conditional cooperators are *explicitly* cooperating due to the experimental set-up, whereas reciprocators are potentially reciprocating coordination and cooperation. This could indicate that when real-world dilemmas contain a mix of coordination and cooperation dilemmas, reciprocity tends to be even more of an evolutionarily stable strategy in the long-term facilitation of general prosociality.

*Group variation*. Finally, we find that buying clubs vary dramatically in their reciprocal economic behaviour. Clubs vary in the amount of reciprocity they exhibit on average (from 91.7 to 59.7%), and in their member behavioural-type composition (16.7 to 100% reciprocators, 0–33.3% beneficiaries, and 0–81.3% helpers). Group-level variation is a natural phenomenon in all social contexts; however, research suggests that human cultural (and organizational) evolution may be largely driven by the ability of groups to maintain effective patterns of group cooperation towards collective goals (Richerson et al., 2016; Waring et al., 2022). Thus, to the extent that the differences we observe in economic reciprocity are indicative of underlying patterns of cooperation, they may be consequential in the survival of these small clubs. Further research is required to investigate the consequences of reciprocity on club persistence.

The wide variation we observe could be evidence that these clubs use multiple means of solving social dilemma. Clubs with a higher abundance of beneficiaries and helpers could be structured that way because helpers gain utility both from the items they purchase and from assisting other members of the club, i.e. they have a social preference that is fulfilled by their club’s success. This is in line with survey evidence where some members have indicated that they enjoy completing bulk purchases that would otherwise go unfilled (Hupper, 2017, p. 1). Moreover, clubs could establish norms and institutions as they age to reduce the cost of their social dilemma or eliminate it entirely.

*Proof of concept*. The greatest implication of our research is that patterns of cooperation in real-world situations are increasingly observable thanks to digital records of economic interactions. Human cooperative behaviour has long matched qualitative and ethnographic descriptions of human behaviour (Henrich & Muthukrishna, 2021). However, behavioural games that measure cooperation, such as the PGG or the DG, can now be more directly calibrated to true economic cooperation. Furthermore, our results highlight that altruism, cooperation, and coordination operate within a spectrum of prosocial behaviour, and reciprocity is an effective strategy at maintaining them all. As such, researchers intent on understanding human cooperation in a naturalistic setting must further integrate real-world scenarios into models of human prosociality. Our analysis shows that this is not only possible, but necessary for the study of cooperation.

The differences between the structure of co-purchases and experimental public goods settings are instructive in this regard. To begin with, members of these clubs are part of a group with clear, narrow goals that substantially overlap with one another, that is, an organization. In experimental settings, individuals are often strangers with no previous affiliation (though the findings of Smith et al. (2018) come from hunter-gatherers who are affiliated tribe members). Many studies of cohesive groups that manage resources have qualitatively deduced high degrees of prosociality (Ostrom, 1990; Wilson et al., 2013), and survey evidence reveals that members of these clubs are similarly non-random and self-selected (Hupper, 2019; Lange, 2022; Tremblay and Waring, 2015), so we would expect them to be more cooperative and reciprocal than strangers in a game because of their stake in the club itself and their familiarity with their clubmates. Consequently, our results serve as a quantitative verification that individuals with established relationships in organizational contexts express greater levels of reciprocity and cooperation than would be expected based solely on laboratory experiments with strangers.

That these clubs are made of up of non-random individuals prosocially combining their purchase power to supply a missing market reveals how our results probably generalize most to situations where traditional market mechanisms are missing. The history of consumer co-operatives is rife with examples where individuals pool their resources to make markets going all the way back to the beginning of the co-operative movement (Waring et al., 2022). Adjacent organizations committed to mutual aid have been supplying services and information to underserved populations for decades, including by providing medical and mental health services (Archibald, 2007) and by forming temporary hubs to reduce food waste and address food insecurity during COVID-19 (Lofton et al., 2022). Informal and pre-formal organizations and networks that rely on prosociality probably rely on high rates of reciprocity as well, at least until formal rules can be established.

A final key difference between food-buying clubs and experimental settings is that individuals in PGGs receive tangible monetary benefits through uniform pay-offs (plus the amount they kept during donation). In food clubs, individuals do not receive a uniform share pay-off, but gain a discount on the goods they purchased in bulk as well as access to a greater selection of goods that may not be found in traditional market settings (i.e. the grocery store) by being in the club. This reveals that in this real-world setting, individuals act reciprocally even though the benefits are not necessarily monetary (access to the club and intangible utility from items) and are heterogeneous across individuals (as discounts are different depending on what items are bought).

*Limitations and future work*. There are a few limitations in our study. First, a nuanced time-series analysis would provide a better understanding of stability, though many of these models are infeasible when club orders occur at irregular intervals. There are also aspects of reciprocity and cooperation that our share-based and purchase-order-based criteria of assistance do not capture. For example, there may be situations where demand exceeds the threshold for an item but is insufficient to purchase two or more cases. While the first case will be fractionally Pareto optimal, it still does not reach the *actual* optimum. In this circumstance, we may observe reciprocity based on share if some individuals step up to fill more than one case, but we would not observe it if individuals accept less than their ideal amount to allow access to all who wanted some. Furthermore, as stated before, our purchase-order-based criterion is potentially confounded by unobservable factors such as internet access and individual schedule that may arbitrarily affect when individuals are able to pledge their share.

A potential drawback of our method of calculating reciprocity is that it may not fully account for the utility costs associated with how prosociality can be conceptualized within these groups. To illustrate, we can imagine a situation where an individual’s lions share of a co-purchase is more (or less) than their optimum, while those with smaller shares are purchasing at their optimum. Though it is still justifiable to say that those who buy less are still helping the member with the most to avoid failing to purchase, we would not pick up the cooperative nature of the utility cost being assumed by the principle buyer so the secondary buyers do not have to deviate from their optimum. This situation would result in us *under-counting* the true amount of reciprocity and the degree of cooperation involved. Our results may therefore represent a conservative estimate of reciprocity.

The example above highlights how difficult it can be to truly disentangle prosocial behaviours from each other in naturalistic settings, despite quality data. While we are confident that our singular assistance edge definition comes close to matching the cooperation and altruism elicited by laboratory studies, further study that incorporates other factors would refine our estimates. Future studies might fit dynamical models that measure individual preferences over time to determine how much of the observed reciprocity is experienced as altruistic. Additionally, future surveys of members could elicit how often they buy more or less of an item than preferred on behalf of others, as well as how often they feel their shares sacrifice their ability to purchase goods they might have otherwise preferred. This would allow us to better quantify the extent to which reciprocity can be portioned into sustaining coordination, cooperation, and altruism.

### 5.1. Summary

We have used a novel approach to detect and describe reciprocity in the economic networks of small food-buying clubs in which economic cooperation is thought to be necessary. Our observational data validate three key findings of the prior experimental literature on cooperation and extend the domain in which we can expect cooperation and reciprocity to be applicable. We find higher levels of reciprocity compared to previous studies of altruism, cooperation, and coordination. We find similar fractions of behavioural types, with reciprocators or conditional altruists being most common. We find that rapid dyadic reciprocity is much more common than indirect reciprocity and that short-term reciprocity is more common than longer-term patterns. The results of our study imply that a notable fraction of economic reciprocity in buying clubs is cooperative, and even altruistic, rather than merely coordinative, because the patterns match those of experimental studies designed to elicit cooperation. Further research might use similar methods to explore the evolution of cooperation in different organizational contexts.

## Supporting information

Lange and Waring supplementary material Lange and Waring supplementary material

## Footnotes

1 We use co-operative with a hyphen to denote organizations that function via a model of user ownership, e.g. co-operative grocery stores that are owned by member shoppers or worker co-operatives that are owned by employees.

## Supplementary material

The supplementary material for this article can be found at https://doi.org/10.1017/ehs.2025.8

## Research transparency and reproducibility interest

All data and code used in this paper are available at https://github.com/tzlange16/ Reciprocity-in-Real-World-Networks.
