# Supplementary material for: Observational evidence that economic reciprocity pervades self-organized food co-operatives
Source: Evol Hum Sci. 2025 Feb 14;7:e12. doi: 10.1017/ehs.2025.8 (PMC12041341; doi:10.1017/ehs.2025.8)
Supplement: Lange and Waring supplementary material [file S2513843X25000088sup001.pdf]

Supplemental Material: Observational evidence that economic reciprocity pervades self-organized food co-operatives

Taylor Z. Lange[abd]* taylor.z.lange@maine.edu
Timothy M. Waring[cd] timothy.waring@maine.edu

[a] Margaret Chase Smith Policy Center, University of Maine, Orono, ME 04469, United States
[b] Ecology & Environmental Sciences, University of Maine, Orono, ME 04469, United States
[c] Mitchell Center for Sustainability Solutions University of Maine, Orono, ME 04469, United States
[d] School of Economics, University of Maine, Orono, ME 04469
*Corresponding Author: taylor.z.lange@maine.edu

Sample Characteristics

The clubs in our data set are samples from WEIRD (Apicella, Norenzayan, and Henrich 2020; Henrich 2020; Henrich, Heine, and Norenzayan 2010) countries: 2 from Australia, 1 from Canada and the rest from the United States. One software provider gave us access to their whole catalog of groups, which included the 3 from outside of the United States, and the other only gave us access to their clubs located in New England. The sample includes defunct and still functioning clubs (as of the end of the data period), where the average lifespan of defunct clubs was 3.16 years (2.16 SD) and 28.7 orders (21.3 SD), while the censored average lifespan for continuing clubs is 3.24 years (1.38SD) and 46.9 orders (27.9 SD). The average number of members participating in an order is 7.96 for defunct clubs (6.96 SD) and 12.7 for ongoing (9.41 SD). Additionally, the average number of co-purchased items in each order is 3.29 for defunct clubs (4.82 SD) and 11.2 for ongoing clubs (28.9 SD).

Reciprocity Accounting

The purchasing process takes place over many days, so members may reciprocate within an order. Direct within-order reciprocity (DWR) in each order t is the number of mutually extended edges within each dyad. This can be found by taking the minimum dyadic out-degree between each partner i and j of a dyad. For example, if member i assists member j with 4 bulk purchases, and member j assists member i with 3 bulk purchases, then DWR$=3$ for i and j, and 6 for the dyad $(i+j)$. We then remove these matched edges from the network to prevent double counting when assessing direct between-order reciprocity (DBR), which is calculated by matching remaining mutually extended edges across many orders for each dyad (i.e., matched edges from order t and $t+1$, $t+2$, etc.). Matched edges on each dyad are recorded and removed from the networks at each time step so that they cannot be double counted when assessing indirect reciprocity.

Once direct reciprocity is accounted for, we look, again, at individual orders to tally indirect within-order reciprocity (IWR). Here, we match any non-dyadic in- and out- edges per individual. For an individual i at time t this is calculated as the minimum of their in- and outdegree. As with direct reciprocity, these matched edges are removed from each order network to calculate indirect between-order reciprocity (IBR) by matching any remaining edges in order t with the remaining edges on each future order's network ($t+1$, $t+2$, etc.). These matched edges are again removed from the network, leaving only unreciprocated edges (UR). However, these unreciprocated edges have the potential to become reciprocated in future orders, so these counts should be treated as simultaneously unreciprocated and potentially reciprocated.

	**Direct Reciprocity**	**Indirect Reciprocity**
**Within Order**	$DWR_{i,j,t} = min(k_{i,j}^{out}, k_{j,i}^{out})$	$IWR_{i,t} = min(k_{i,t}^{out}, k_{i,t}^{in})$
**Between Orders**	$DBR_{i,j,t} = min(\sum_t^T k_{i,j,t}^{out}, \sum_t^T k_{j,i,t}^{out})$	$IBR_{i,t} = min(\sum_t^T k_{i,t}^{out}, \sum_t^T k_{i,t}^{in})$

Table 1: Reciprocity is calculated by counting paired edges across co-purchasing networks. Social and temporal proximity is given precedence in counting paired edges, so that more proximate interactions are removed before counting more distant interactions, producing the following order: DWR, DBR, IWR, IBR. Finally, remaining unmatched co-purchasing edges are counted as unreciprocated, UR.

References

Apicella, Coren, Ara Norenzayan, and Joseph Henrich. 2020. "Beyond WEIRD: A Review of the Last Decade and a Look Ahead to the Global Laboratory of the Future." *Evolution and Human Behavior* 41: 319–29. https://doi.org/10.1016/j.evolhumbehav.2020.07.015.

Henrich, Joseph. 2020. *The WEIRDest People in the World: How the West Became Psychologically Peculiar and Particularly Prosperous*. Farrar, Straus; Giroux.

Henrich, Joseph, Steven J. Heine, and Ara Norenzayan. 2010. "The Weirdest People in the World?" 33: 61-83; discussion 83-135. https://doi.org/10.1017/S0140525X0999152X.

Table 2: Share-based Reciprocal Edge Classifications and Coefficients of Variation

			Percent of Total Edges				Coefficient of Variation			
c_number	Orders	Members	DWR	DBR	IWR	IBR	DWR	DBR	IWR	IBR
1	59	23	70.87	11.97	3.28	2.80	28.77	78.05	120.88	147.78
2	11	10	64.71	0.00	5.88	0.00	73.43		331.66	
3	22	28	67.22	7.53	5.51	5.33	19.21	91.81	87.29	112.77
4	27	18	61.61	7.59	6.70	6.47	68.37	154.02	137.39	172.58
5	69	9	57.27	14.98	2.64	5.73	63.31	147.32	281.49	242.32
6	15	4	63.06	14.41	4.50	6.31	81.21	146.99	273.94	230.75
7	93	8	63.72	15.93	1.77	6.64	79.24	150.72	450.82	271.84
8	64	66	54.21	15.11	10.31	4.17	43.17	109.03	93.08	172.86
9	48	26	45.65	11.68	10.05	7.07	59.34	152.03	132.13	216.36
10	91	122	57.66	11.87	9.91	4.06	13.53	40.11	39.79	86.51
11	67	31	58.35	12.01	6.84	4.10	43.81	91.61	109.77	135.80
12	10	27	71.52	11.17	6.59	2.49	9.65	37.65	28.08	56.07
13	69	51	58.29	14.67	6.88	4.90	27.74	55.13	56.51	96.41
14	80	46	55.42	17.70	7.50	4.70	33.05	75.29	102.94	103.74
15	80	144	52.23	10.25	11.01	6.93	24.58	51.12	52.48	58.61
16	76	78	57.64	13.33	8.57	4.06	22.30	49.65	58.45	92.28
17	30	9	55.06	22.12	4.24	4.24	41.59	59.62	160.40	175.02
18	46	6	55.56	6.35	2.38	6.35	62.84	173.14	368.19	227.58
19	20	3	67.92	15.09	0.00	5.66	83.63	183.90		324.63
20	14	9	57.76	20.05	3.10	2.86	21.63	49.64	136.29	138.62
21	52	66	61.26	15.35	6.88	2.87	12.20	35.95	48.67	63.86
22	95	66	66.18	14.14	5.39	1.16	23.07	51.71	55.79	393.82
23	100	141	73.70	7.93	5.85	1.64	22.59	52.21	72.25	296.70
24	34	45	63.69	8.09	8.91	1.86	6.35	37.19	23.71	75.64
25	30	80	64.49	6.05	9.31	1.58	8.67	37.68	24.51	88.82
26	5	7	54.17	4.17	6.25	6.25	40.61	142.88	100.33	189.81
27	34	7	71.70	7.55	1.26	6.92	52.25	198.15	329.63	245.41
28	17	23	61.07	14.18	6.08	7.09	20.82	54.33	99.73	98.16
29	67	34	61.24	19.19	4.86	3.76	19.04	49.68	77.84	94.82
30	17	4	66.67	0.00	0.00	9.52	55.08			209.44
31	44	7	36.36	33.77	5.19	15.58	112.18	102.91	277.17	158.48
32	37	7	60.13	6.54	3.92	3.27	70.79	168.90	215.30	270.34
33	5	11	47.04	0.00	14.88	1.12	7.86		25.75	124.32
34	6	6	61.29	8.76	3.92	0.00	16.16	89.78	78.31	
35	62	16	44.50	3.46	11.01	0.71	19.25	90.96	61.45	296.87
**Totals**										
	1596	1238	59.69	11.51	6.04	4.52	39.64	94.04	136.73	171.79

Table 3: Purchase Order-based Reciprocal Edge Classifications

	Percent of Total Edges			
Club Number	DWR	DBR	IWR	IBR
1	23.8	40.8	8.6	15.3
5	15.4	28.4	7.1	29.0
7	8.3	27.4	5.4	8.9
8	15.7	35.0	19.8	13.6
18	18.9	21.1	10.5	44.2
19	11.4	51.4	2.9	28.6
20	29.9	38.2	5.1	10.7
22	43.4	25.4	9.7	13.0
27	24.1	38.9	2.8	27.8
29	28.8	29.7	10.5	14.6
30	0.0	14.3	21.4	64.3
31	6.2	56.2	3.1	28.1
32	17.4	20.9	9.6	35.7
**Means**				
	18.7	32.9	9.0	25.7

Table 4: Mixed Effects Models

	Dependent variable:		
		Out Degree	
	All Edges	Singular Assistance Edges	Robustness Check
	(1)	(2)	(3)
Global Mean Intercept	25.936***	4.006***	80.588
	(9.869)	(1.199)	(60.302)
In Degree	0.882***	0.457***	0.635***
	(0.009)	(0.011)	(0.026)
Random Effects:			
Standard Deviation	36.36	6.71	169.67
Clubs	35	35	13
Observations	1530	1113	263
REML	21098.26	8248.94	3949.23
AIC	21106.26	8256.94	3957.23
Note:			*p<0.1; **p<0.05; ***p<0.01

Table 5: SPR Cluster Centers

c_number	Beneficiary	Reciprocator	Helper
1	-0.880	0	0.836
2	-0.742	0	0.783
3	-0.977	0	0.868
4	-1.160	0	0.905
5	-1.033	0	0.706
6	-0.805	0	0.766
7	-0.730	0	0.864
8	-1.033	0	1.037
9	-1.053	0	0.956
10	-1.403	0	0.816
11	-1.059	0	0.948
12	-1.707	0	0.467
13	-1.125	0	0.904
14	-1.062	0	0.873
15	-1.180	0	0.904
16	-1.083	0	0.871
17	-0.888	0	0.878
18	-0.904	0	0.761
19	-0.573	0	0.678
20	-0.970	0	0.707
21	-1.063	0	0.695
22	-0.885	0	0.745
23	-1.527	0	0.537
24	-0.999	0	0.688
25	-1.633	0	0.586
26	-0.981	0	0.798
27	-0.621	0	0.733
28	-0.922	0	0.731
29	-0.952	0	0.864
30	-1.134	0	0.505
31	-0.952	0	0.747
32	-0.920	0	0.709
33	-1.969	0	0.663
34	-0.761	0	0.847
35	-1.527	0	1.007

Table 6: Count of Member Types

c_number	Beneficiaries	Reciprocators	Helpers	Unclassified*
1	0	21	2	4
2	2	8	0	2
3	3	22	3	7
4	1	14	3	9
5	1	7	1	1
6	1	3	0	10
7	2	6	0	4
8	7	49	10	11
9	3	19	4	6
10	9	87	26	25
11	3	22	6	6
12	3	19	5	3
13	3	35	13	13
14	5	31	10	10
15	16	97	31	46
16	13	52	13	11
17	1	6	2	3
18	1	4	1	11
19	0	2	1	5
20	1	6	2	1
21	11	43	12	2
22	5	47	14	6
23	10	85	46	41
24	5	27	13	6
25	3	48	29	16
26	1	4	2	3
27	0	4	3	2
28	5	13	5	6
29	3	19	12	5
30	0	2	2	1
31	1	3	3	1
32	1	3	3	3
33	1	4	6	8
34	1	2	3	2
35	2	4	10	2
**Totals**				
	124	818	296	292

Note:
* Members who only participate in one order do not have a Markov matrix and thus remain unclassified
